# Ultrasound-Propelled Nanocups for Drug Delivery

**DOI:** 10.1002/smll.201501322

**Published:** 2015-08-21

**Authors:** James J Kwan, Rachel Myers, Christian M Coviello, Susan M Graham, Apurva R Shah, Eleanor Stride, Robert C Carlisle, Constantin C Coussios

**Affiliations:** Institute of Biomedical Engineering, University of OxfordOxford, OX3 7DQ, UK E-mail: constantin.coussios@eng.ox.ac.uk; Department of Oncology, University of OxfordOxford, OX3 7DQ, UK

**Keywords:** cancer therapy, cavitation, drug delivery, nanoparticles, ultrasound

## Abstract

Ultrasound-induced bubble activity (cavitation) has been recently shown to actively transport and improve the distribution of therapeutic agents in tumors. However, existing cavitation-promoting agents are micron-sized and cannot sustain cavitation activity over prolonged time periods because they are rapidly destroyed upon ultrasound exposure. A novel ultrasound-responsive single-cavity polymeric nanoparticle (nanocup) capable of trapping and stabilizing gas against dissolution in the bloodstream is reported. Upon ultrasound exposure at frequencies and intensities achievable with existing diagnostic and therapeutic systems, nanocups initiate and sustain readily detectable cavitation activity for at least four times longer than existing microbubble constructs in an in vivo tumor model. As a proof-of-concept of their ability to enhance the delivery of unmodified therapeutics, intravenously injected nanocups are also found to improve the distribution of a freely circulating IgG mouse antibody when the tumor is exposed to ultrasound. Quantification of the delivery distance and concentration of both the nanocups and coadministered model therapeutic in an in vitro flow phantom shows that the ultrasound-propelled nanocups travel further than the model therapeutic, which is itself delivered to hundreds of microns from the vessel wall. Thus nanocups offer considerable potential for enhanced drug delivery and treatment monitoring in oncological and other biomedical applications.

## 1. Introduction

Despite the leaky vasculature that is characteristic of tumors,[1] current anticancer therapeutics such as small molecule drugs,[2] antibodies,[3] drug-loaded liposomes,[4] and oncolytic viruses[5] are unable to penetrate further than approximately 50 μm beyond a blood vessel and into hypoxic cancerous tissue. As a result, poor drug distribution has been identified as one of the primary factors limiting cancer therapy.[6,7] This inability to achieve sufficient tumor penetration is in part due to excessive reliance on the enhanced permeability and retention (EPR) effect for passive accumulation of the therapeutic agent. There has thus been a push in developing techniques, such as mechanically activated transport mechanisms[8] and light,[9] pH,[10] or chemically[11] triggered drug release, in order to promote drug distribution. Recently, it has been shown that remote mechanical activation of small gaseous particles with ultrasound enables penetration of an oncolytic virus into a solid tumor by hundreds of micrometers, compared to tens of micrometers in the absence of ultrasound.[5]

Gas bubbles expand and contract in response to the positive and negative pressure phases of an ultrasound wave. This dynamic behavior is known as cavitation. Uncontrolled expansion of a bubble under certain conditions reduces the pressure inside the cavity until the gas–liquid interface is unable to support the inertia of the surrounding liquid and the cavity collapses violently.[12–14] Microstreaming associated with such cavitation activity[15] has been shown to promote the penetration and extravasation of free drugs for a variety of applications.[16–18]

In the context of drug delivery for cancer, such active transport may provide higher and more rapid levels of tumor accumulation than that afforded by EPR. To date, all studies seeking to mechanically enhance drug delivery to tumors have utilized shelled microbubbles, approved for clinical use as diagnostic ultrasound contrast agents.[19,20] However, at the ultrasound amplitudes required to enhance drug delivery,[5,21,22] the comparatively large microbubbles (2–10 μm in diameter)[22,23] are destroyed by exposure to ultrasound, thus limiting their capacity to enhance drug penetration. Ultrasound parameters have also been adjusted to allow sufficient reperfusion of micro­bubbles into the tumor volume.[24] Yet even with these advances, substantial cavitation activity from microbubbles is typically sustained for less than 30 s.[23,25,26] This is incompatible with the need to enhance the delivery and tumor penetration of drugs, which typically circulate for tens of minutes. Thus multiple injections of microbubbles are required to sustain cavitation-enhanced drug transport. Even though this is possible in small animal models, the need for repeat injections or continuous infusion would exceed the maximum allowable dose for these agents in humans (0.06 mL kg^−1^).[27] This typically amounts to a maximum of 2 mL per injection and a maximum of two injections per patient per visit.

**Figure 1:**
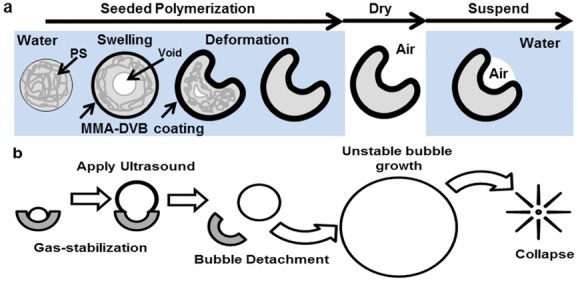
Schematics of the formation of nanocups and a proposed mechanism for nucleating cavitation are shown. (a) Nanocups are produced by a seeded polymerization technique whereby a template nanoparticle comprised of polystyrene (PS) is coated with MMA with divinylbenzene (DVB) as the cross-linker that induces swelling and deformation of the template. After the formation of the “cup,” the nanoparticle suspension is dried. Upon resuspension, the nanocups trap gas within the cavity. (b) The nanocup with nanobubble construct is activated upon exposure to ultrasound. During the rarefactional pressure phase of the ultrasound wave, the nanobubble grows and detaches from the cavity. Once free, the bubble continues to grow uncontrollably until the compressional phase of the ultrasound, which causes the bubble to collapse.

To address this limitation, we have developed a novel solid–gas nanoparticle that can sustain cavitation activity for several minutes at ultrasound pressure amplitudes generated by conventional diagnostic transducers as well as therapeutic ultrasound systems. In order to achieve this, a biocompatible cup-shaped nanoparticle, henceforth referred to as a nanocup, was designed to entrap a surface nanobubble that detaches and collapses upon ultrasound exposure. We have extensively modified an interfacial seed polymerization method (**Figure** **1**a) to achieve the formation of cup-shaped nanoparticles for the very first time. Following formulation and characterization, an air-drying and resuspension procedure was further developed and optimized to capture and stabilize a nanobubble within each nanocup without affecting their size, shape, or tendency for agglomeration. Upon ultrasound exposure, the stabilized nanobubble nucleates a cavitation event (Figure 1b) that enables active micropumping of the surrounding fluid and of any agents suspended within it. In vitro and in vivo measurements were then made to quantify the cavitation activity and associated cavitation-enhanced drug transport. Specifically, cavitation activity was imaged in real time using a novel ultrasound technique known as passive acoustic mapping (PAM),[28–31] indicating that nanocups circulate well, and cavitate reliably and for considerably longer than microbubbles following intravenous injection in an in vivo tumor model. Cavitation activity associated with the nanocups resulted in enhanced distribution of IgG mouse antibody throughout a CT-26 tumor model, one of the most commonly used models for drug delivery. Because quantification of the enhanced delivery and distribution of a therapeutic relative to blood vessels is difficult in vivo, an in vitro tissue model was further developed to fully characterize the transport enhancement offered by ultrasound-activated nanocups compared to microbubbles and conventional non-ultrasound-based passive delivery. The model shows significantly enhanced extravasation of a model drug in the presence of nanocups and ultrasound. The nanocups themselves also penetrate well beyond the vessel wall, in some cases to distances greater than the molecular drug.

## 2. Results and Discussion

### 2.1. Nanocup Characterization

Composition, shape, and size are critical design parameters for nanoparticles activated by ultrasound that are intended to be used for applications in cancer diagnosis and therapy. Taking these design criteria into consideration, we extensively modified and optimized a seeded polymerization technique in order to coat a polystyrene template nanoparticle with a cross-linked polymethyl methacrylate polymer in order to achieve formation of a nanocup of a suitable aspect ratio to stabilize a nanobubble (Figure 1a).[32] It is worth noting that both of the monomer constituents used already featured on the list of inactive ingredients of FDA-approved drug products and of FDA-approved color additives exempt from certification.

The seeded polymerization method has never been previously used to produce either nanocups or ultrasound-responsive gas-entrapping nanoparticles. In order to achieve the latter, an optimized air-drying technique described in the Experimental Section was used to trap gas within the cavity of the nanocup upon resuspension.

**Figure 2:**
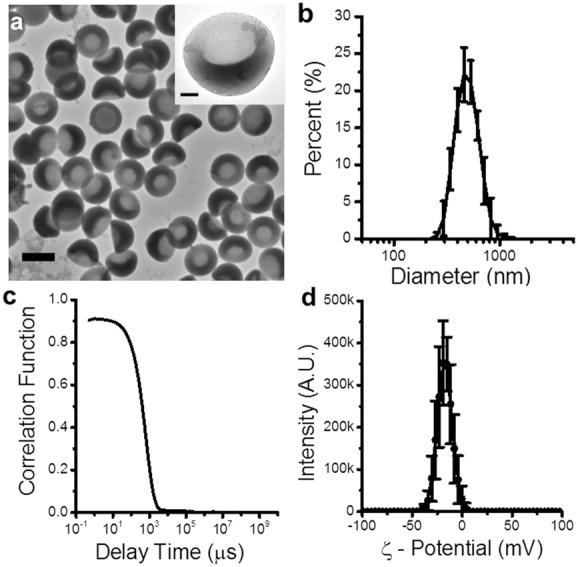
Physical characterization of nanocups demonstrates their uniform shape and size. a) TEM images of representative nanocups. The scale bar represents 500 nm. The inset is a TEM image of a single nanocup, emphasizing the “cup” shape of the nanoparticle. The scale bar represents 100 nm. b,c) DLS size distribution and corresponding correlation function of the nanocups, respectively. d) Surface charge measurements of nanocups.

When the samples were imaged with transmission electron microscopy (TEM), it was evident from the different settling orientations that nanocups displayed a definitive uniform “cup” profile (**Figure** **2**a) with cavity diameters between 230 and 340 nm. This depression is formed during the polymerization of the copolymer shell and its uniformity is dependent on the degree of crosslinking within the shell. The transition from sphere to “cup” shape is due to the poly­mer–polymer interactions between the polystyrene core and copolymer shell.[33] These interactions cause the core to swell, while osmotic forces bend the shell to form the depression.[32] The crosslinking of the shell changes its rigidity, allowing it to be pliable enough to bend, but stiff enough to prevent multiple depressions from forming.

The size distribution of the nanocup suspension (Figure 2b) was determined using dynamic light scattering (DLS). Nanocups showed a consistent hydrodynamic diameter of 480 ± 24 nm. It is crucial to note that the DLS correlation function (Figure 2c) decreased to zero, which verified that the suspensions were not contaminated with micro­meter-sized agglomerations. Such stringent validation was essential to prevent inertial cavitation events being falsely ascribed to macro-scale contaminants.[34] The lack of agglomeration is due to the electrostatic repulsion forces caused by the negative zeta potential (>−20 mV) of the nanoparticles (Figure 2d).

### 2.2. Tuning Nanocup Size and Ultrasound Response

**Figure 3:**
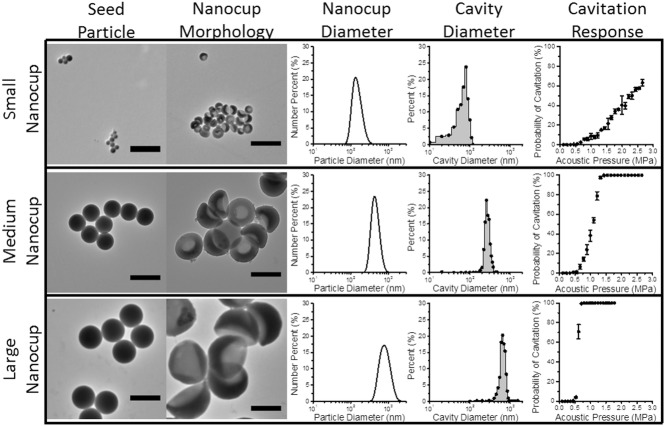
Tunability of nanocups is shown alongside its influence on cavity size and cavitation response. TEM images of small (100 nm), medium (300 nm), and large (460 nm) seed particles control the diameter and cavity size of the nanocups (that are categorized based on the seed particle diameter). Furthermore, cavity diameter affects the pressure amplitude required to nucleate from cavitation.

By using seed particles of different diameters, we were able to manufacture nanocups with distinct cavity sizes of 180, 260, and 600 nm in diameter (**Figure** **3**). It is understood that the pressure amplitudes required to nucleate cavitation from a bubble within a cavity is proportional to cavity diameter.[35] Therefore we measured the acoustic response of nanocups with different cavity mean diameters exposed to ultrasound with a center frequency of 0.5 MHz at a range of pressure amplitudes. From Figure 3, the nanocups with the smallest cavity diameter required pressure amplitudes between 2.5 and 3 MPa to nucleate cavitation with some reliability. In contrast, the nanocups with medium sized cavity diameter (260 nm) were able to achieve consistent cavitation response at pressure amplitudes between 1 and 1.5 MPa. The nanocups with the largest cavity diameter required only 0.5 MPa to nucleate cavitation. It was evident that there was a correlation between nanocup cavity diameter and ultrasound response, which corroborates prior work.[35]

There are two critical requirements in choosing the most suitably tuned nanocup for drug delivery in tumors. First, the pressure amplitudes needed to nucleate cavitation (that increases monotonically with driving frequency) must not induce harm from off target cavitation events. From this requirement, it is clear that the smaller nanocups are suboptimal for enhancing drug delivery. Second, we want to maximize the quantity of cavitation nuclei for a given dose of nanocups in order to optimize the ultrasound response from the nanocups. Nanocups with a medium cavity diameter have roughly 10 times more particles than nanocups with a large cavity diameter for the same given dose. Therefore we have chosen to use nanocups with a medium cavity size for the rest of the experiments as it satisfies both requirements.

### 2.3. Biodistribution and Circulation of Nanocups

Preliminary pharmacokinetic and biocompatibility studies were carried out for injections of 0.3 mg and 0.6 mg of nanocups in a murine model. As shown in Figure S2 (Supporting Information), in the absence of ultrasound exposure, some 35% of the higher injected dose (0.6 mg) was found to accumulate in the liver within 5 min of administration, but less than 10% of the injected dose remains in the liver 2 h after administration. Analysis of the fluorescence associated with the urine indicated that this was the primary route of excretion. However, it is presently unclear whether this relates to the fluorophore alone or all the components of the cups. In previous reports, hepatic metabolism and urinary and fecal excretion have been evidenced for these divinylbenzene (DVB) (the crosslinking agent) and poly(methyl methacrylate) (the primary component of the nanocup surface).[36,37] However, further experimental work will be needed to clarify and confirm the clearance pathway and rate suggested by these preliminary experiments.

**Figure 4:**
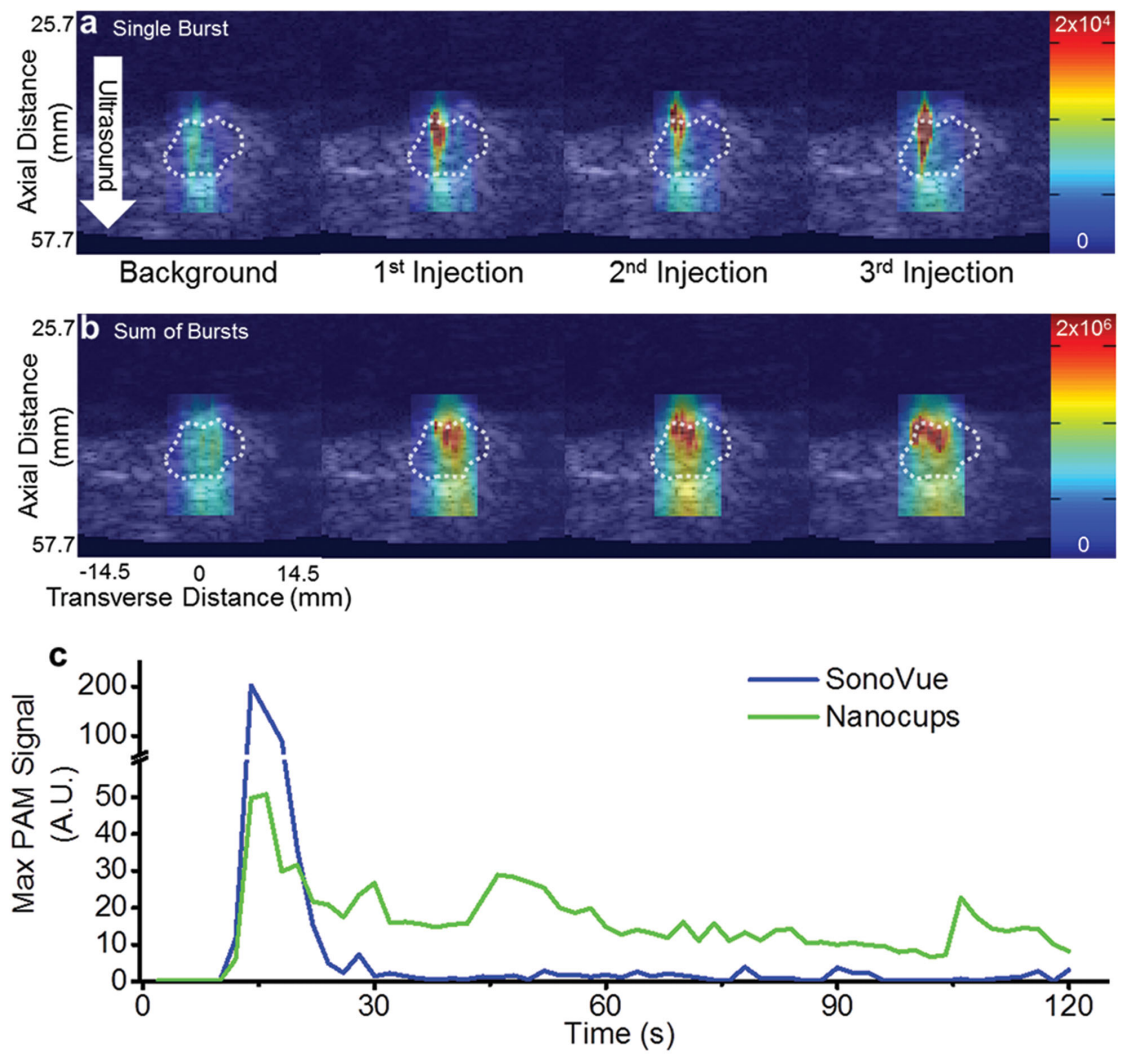
Representative images of inertial cavitation events within the tumor. a) Single-frame stills of a B-mode image with a PAM overlay. PAMs were generated during the ±3° acoustic sweep during the background injection and after each of the three injections. b) Summed PAM overlay of the entire acoustic sweep. In all of the images, the dotted white line represents the edge of the tumor (determined by a blind observer). The color bars represent arbitrary units proportional to power. A white arrow demarcates the direction of the ultrasound. The axial and transverse distances are valid for all images. c) Representative maximum PAM signals from a tumor after intravenous injection of SV or nanocups and exposure to ultrasound (0.5 MHz, 1.5 MPa).

The pharmacokinetic data (Figure S2, Supporting Information) indicates that over 15%–20% of the initial nanocup dose remains in the bloodstream 20 min after intravenous administration. This provides an adequate time window to cause cavitation in tumors and potentially enhance the delivery of a coadministered therapeutic. Such duration is substantially longer than microbubbles, which clear within 2 min of injection, as illustrated in **Figure** **4**. Furthermore, 20 min circulation in a mouse is likely to translate into 100–200 min in a human due to the greatly increased circulatory rate of the mouse.

Intravenous administration of up to 0.6 mg of nanocups (the amount used to induce cavitation in tumors) did not produce an increase in C3a concentration, interleukin-6 concentration, alanine aminotransferase, and tumor necrosis factor-α above that found in an injection of a buffer solution (data not shown). No overt signs of toxicity were observed in injected mice with activity, socialization, and weight remained unaltered for up to 20 days following administration of nanocups.

### 2.4. Monitoring Cavitation Activity from Nanocups in Tumors

We next investigated the ability to nucleate cavitation from nanocups in a tumor using a diagnostic ultrasound imaging system, in order to move towards developing a readily applicable enhanced cancer therapy strategy. Female BALB/c mice were subcutaneously implanted with CT-26 cells, and the tumors were allowed to grow to up to 300 mm^3^. Nanocups were injected via a tail vein cannula and exposed to ultrasound using a conventional diagnostic probe (C5-2 128 element probe with a 2 MHz center frequency) to deliver a custom pulse sequence of interlaced low-amplitude imaging pulses with high-amplitude therapy pulses. The low-amplitude pulses were received and reconstructed into B-mode images whereas the spatially focused high-amplitude pulses (4.5 MPa) activated the nanocups. The resulting broadband signals from the cavitating nanocups were received and reconstructed into passive acoustic maps (PAMs)[28–31] which can be overlaid onto the B-mode images. Figure 4a shows representative single-frame stills of a B-mode image of the tumor with a PAM overlay that was generated by a high amplitude ±3° acoustic sweep after background injection and each of the three nanocup injections. In each case, the “background” injection was performed with the suspending medium only (in the absence of nanocups), which demonstrated no significant cavitation events. Only upon injection of nanocups were significant amounts of inertial cavitation activity detected with PAM. Furthermore, it was apparent that after subsequent injections, there was more cavitation activity. This accumulation effect was most pronounced in Figure 4b, which displays the summed PAMs over all of the frames (representing the entire 1 min long collect period), demonstrating that the cavitation was both more intense and covered more of the tumor after each injection. Moreover, these images indicated that the location of inertial cavitation was isolated to the periphery of the tumor, a region that is highly vascularized. Video S1 (Supporting Information) is a representative steered sweep on a single mouse.

Across the three injections of the three mice, there was an observed increase in received cavitation power (Figure S1, Supporting Information). One possible reason for this increase is that after each injection, the different clearance pathways become saturated. As a result, there becomes an increasing amount of nanocups in the circulating blood. Alternatively, the increased cavitation response may be a result of both passive and active, ultrasound-mediated accumulation of the nanocups into the tumor. Because a majority of nanocups are between 200 and 500 nm (Figure 2b), there is a possibility that a portion of the nanocups are propelled out of the vasculature due to the mechanical mixing effects of cavitation. However, further in vivo studies looking at distribution of nanocups are required to elucidate which of these mechanisms are the root cause for the apparent accumulation and subsequent increase in cavitation activity within the tumor.

We next compared the duration of cavitation response of clinically used microbubbles (SonoVue; SV; Bracco, Italy) and nanocups in a subcutaneous xenograft SKOV-3 tumor. Each sample of cavitation nuclei were injected via a cannula through the tail vein. After a bolus injection of cavitation nuclei, we immediately exposed the tumor to ultrasound (0.5 MHz, 1.5 MPa at 0.5 Hz PRF) for at least 120 s while simultaneously generating PAMs using a similar conventional ultrasound probe (L11-4v with a center frequency of 4 to 11 MHz). Figure 4c shows a representative curve of maximum PAM signal from SV and nanocups throughout the ultrasound exposure time. As expected, SV persists for less than 30 s due to clearance mechanisms and microbubble destruction from the ultrasound beam. In contrast, nanocups sustained a substantial degree of inertial cavitation for the entire 120 s—a period of time four times longer than produced by microbubbles, and any other known cavitation nuclei to date.

The greatly sustained cavitation response exhibited by nanocups is in sharp contrast to the behavior of shelled microbubbles, which are typically destroyed by inertial cavitation in under a minute.[38] Such a rapid depletion effect reduces the efficacy in cavitation-enhanced therapeutic strategies. The continual cavitation activity from nanocups may overcome such a limitation. For example, sustained cavitation is particularly ideal for cancer therapies where therapeutic agents are often continually infused. While many biologics such as oncolytic viruses only circulate effectively for a few minutes, conventional small molecule chemotherapeutics and antibodies in current clinical use have circulation times well in excess of 10 min. It is therefore desirable to cause sustained inertial cavitation for as long as there is drug circulating in order to facilitate its active transport from the blood stream into the target tissue. To meet such requirements, microbubble-enhanced therapies involve repeated reinjections that rapidly exceed the maximum allowable dose recommended by existing manufacturers. By contrast, it is possible that nanocups are capable of delivering sufficient cavitation activity in a single injection or a coinfusion of a therapeutic agent and nanocups. The ability to maintain viable cavitation nuclei for extended periods of time has several implications. It implies that enough viable nanocups are in circulation despite the various innate clearance mechanisms. These results also suggest that a single continuous infusion of nanocups will provide continuous cavitation to enhance active transport of most cancer therapeutics over their circulation time.

### 2.5. Drug Delivery with Nanocups in a Tumor

**Figure 5:**
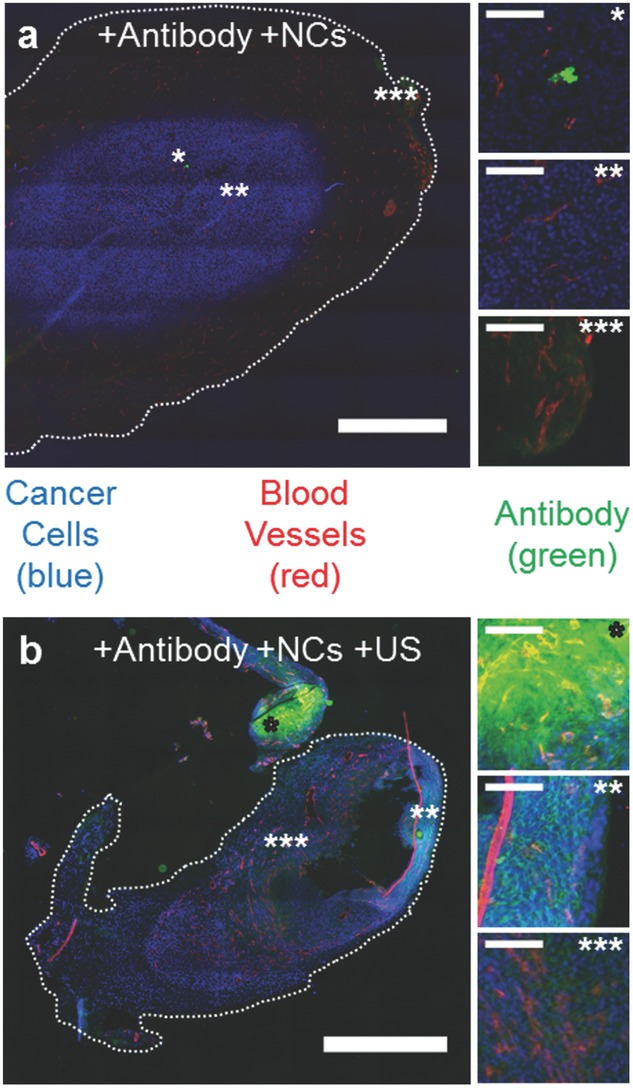
Drug delivery with ultrasound and nanocups (NCs). a,b) Representative fluorescent microscope images of CT-26 tumors treated with IgG antibody (green) and nanocups without (a) and with (b) US exposure (0.5 MHz and 1.5 MPa). Scale bar represents 1 mm. The tumor cells are stained blue and the blood vessels are stained red. The images marked by *, **, and *** show varying amounts of antibody inside the tumor (scale bar is 100 μm for all images) for both (a) and (b). The corresponding location of these images are marked on figures (a) and (b) with the respective number of *'s.

Given that these nanocups are capable of generating cavitation in the tumor, we next conducted a proof-of-concept study to demonstrate their ability to enhance the delivery of a freely circulating model therapeutic not bound to the nanocups in a xenograft tumor. Subcutaneous xenograft CT-26 tumors were treated with a coinjection of a fluorescent IgG mouse antibody and nanocups without and with ultrasound exposure (**Figure** **5**a,b, respectively). Blood vessel endothelial cells were stained with anti-CD31 (red) to show the location of blood vessels. Similarly, the nucleus of the tumor cells was stained with 4′,6-diamidino-2-phenylindole (DAPI) (blue) to indicate the location of the tumor. The IgG mouse antibody was labeled with fluorescein (green). Though it is possible to label the nanocups, they were not used for the study in order to prevent overlaps in fluorescence between the tumor cells and the labeled nanocups.

As shown in Figure 5a, without ultrasound-propelled nanocups, antibody fluorescence was minimal and primarily restricted to either within or directly nearby the microvasculature of the tumor. Some, albeit limited, extravasation was observed in the highly vascularized regions of the tumor. The effect of ultrasound alone was not investigated because previous experimental work in vitro[39] and in vivo,[22] had already demonstrated that no enhanced extravasation of a therapeutic was observed in the absence of any coadministered cavitation agents for the same ultrasound parameters as those used in the present study.

In contrast, in the presence of ultrasound-propelled nanocups in Figure 5b, intensities of antibody fluorescence above background are seen throughout the tumor. In fact, extravasation of the antibody is observed at hundreds of microns from the nearest visible blood vessel. An interesting feature of the zoomed images of Figure 5b is that the antibody resides in the intercellular spaces within the tumor mass.

### 2.6. In Vitro Evaluation of Drug Penetration

Precise quantification of the extravasation and tissue penetration distance of ultrasound-propelled nanocups and the coadministered therapeutic is difficult in vivo. We thus next determined the distribution of the nanocups and model therapeutic after ultrasound exposure in an agarose-based flow phantom. The agarose tissue model chosen has been shown to have similar mechanical properties to soft tissue[40] and a pore size comparable to tumor tissue,[41] namely on the order of 200 to 600 nm. Nanocups were labeled with 9-anthracenylmethyl methacrylate (9ACM) in order to track the final location of the cavitation nuclei after ultrasound exposure. Once labeled, the fluorescent nanocups were mixed with tetramethylrhodamine isothiocynanate dextran (TRITCD), which is a fluorescently labeled sugar with an average molecular weight of 40 kDa simulating a chemotherapeutic. The mixture was coinjected into the tissue model, which contained a single flow channel. Additionally, the model drug alone (i.e., filtered deionized (DI) water without inertial cavitation nuclei) and a mixture of the model drug with SV, a clinically used microbubble suspension, was also tested for their capacity to deliver the model chemotherapeutic.

**Figure 6:**
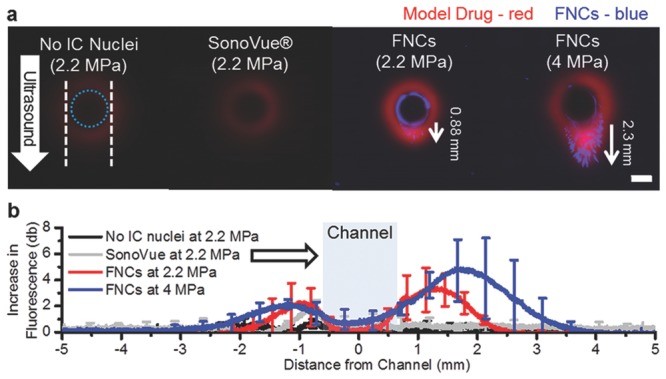
Nanocup penetration in a tissue model. a) Representative fluorescent images of the tissue model sliced radially to the channel after exposure to ultrasound for 5 min at 2.2 and 4 MPa. The white dotted lines indicates the edges of the ultrasound focus, and the dotted blue line in represents the edge of the flow vessel. A white arrow demarcates the direction of ultrasound. In the images, the model drug (TRITCD) is labeled in red and the nanocups are labeled in blue (FNCs). The scale bar represents 1 mm, and is valid for all images. b) Average increase in fluorescence intensity profile plots of no cavitation nuclei, SV, and nanocups at both 2.2 MPa and 4 MPa taken down the center line in the direction of ultrasound are shown for each test condition. The white arrow indicates the direction of ultrasound and the blue box represents the flow channel. TRITCD without cavitation nuclei and without ultrasound exposure was used as the reference value. For clarity, only 20 to 25 points are shown with standard deviations.

As shown in **Figure** **6**a, the fluorescence intensity of the TRITCD in the presence of 2.2 MPa ultrasound and SV microbubbles is comparable to that in the absence of any ultrasound. By contrast, in the presence of nanocups for the same ultrasound pressure (2.2 MPa), there is a significant increase in the TRITCD fluorescence intensity in the perivascular space, to distances in excess of 0.5 mm. This is accompanied by extravasation of the fluorescently labeled nanocups, which exceed 0.8 ± 0.1 mm of penetration distance, particularly in the direction of ultrasound propagation (Figure 6b). Enhanced extravasation of both the TRITCD and nanocups is even more noticeable at 4 MPa, to distances of up to 2.3 mm (with an average distance of 2.1 ± 0.3 mm), with the nanocups greatly exceeding the penetration distance of the model therapeutic. This observation suggests that the nanocups propel themselves under ultrasound exposure, due to the nanobubble entrapped in their surface cavity.

## 3. Conclusion

Previously unreported ultrasound-activated “cup-shaped” polymeric nanoparticles (i.e., nanocups) of mean size 480 nm with polydispersity <0.1 that are capable of trapping and stabilizing nanobubbles were manufactured and characterized. We confirmed that, upon ultrasound exposure, nanocups are capable of seeding cavitation within the tumor environment. Steered focal sweeps of ultrasound showed that the prevalence of cavitation from nanocups primarily located around the perimeter of the tumor, a region that is highly vascularized. PAM also indicated that in addition to strong cavitation events, nanocups may accumulate in the tumor after each subsequent injection. PAM demonstrated that nanocups were capable of providing cavitation for at least 120 s in a SKOV-3 tumor model; in contrast, clinically used microbubbles were depleted in under 30 s. Furthermore, nanocups delivered a substantial amount of fluorescent antibody beyond the microvascular of a CT-26 tumor model and into the intercellular matrix. Nanocups also enhanced the amount and penetration of a coadministered model drug into an agarose soft tissue model of typical porosity 500 nm, to distances well in excess of 800 μm. We speculate that the mechanism for enhanced active transport to be a result of cavitation from nanocups transported into the tissue model. Our results address the current limitations facing cavitation-enhanced cancer therapies by designing a particle whose size will enable tumor penetration. We further demonstrate demonstrating its ability to generate and sustain cavitation activity in order to greatly enhance the transport of molecular therapeutic agents and nanoparticulates in a tumor environment in vivo.

## 4. Experimental Section

*Reagents and Solutions*: Styrene latex beads (PS-LB, 10 wt%) with a 100, 300, and 460 nm diameter, ultrapure agarose, tetramethyrhoadmine isothiocyanate dextran 40 kDa (TRITCD), divinylbenzene (80%, DVB), 2-hydroxyethyl methacrylate (HEMA), methyl methacrylate (MMA), 9-anthracenylmethyl methracrylate (9ACM), fluorescein o-methacrylate (FoMA), potassium persulfate (KPS), glucose, and 4-(2-hydroxyethyl)-1-piperazineethanesulfonic acid (HEPES), were all purchased from Sigma–Aldrich, and used without further purification. SonoVue (SV) was purchased from Bracco UK limited, and reconstituted per ther instructions for use. Deionized (DI) water was made with a Flexeon 300 (GAPS, Lancashire, UK) water system, and filtered (0.22 μm, Fisher Scientific) before use for all nanoparticle formulations.

*Nanocup Preparation*: The nanocups were synthesized by adapting a seed polymerization technique previously described[42] to enable the entrapment and stabilization of gas on PS-LB nanoparticles coated with a copolymer of MMA, HEMA, and DVB. The PS-LB seed particles (2 mL at 10 wt%) were placed into a round bottom flask and diluted with 200 nm filtered DI water until the final volume was 36 mL. Note that for smaller (100 nm) nanocups, only 1.75 mL of PS-LB was used. The flask was de-oxygenated via nitrogen bubbling for at least 30 min. The mixture was placed into a water bath preheated to 80 °C. MMA, HEMA, and DVB were added to the mixture in a 10:1:6 v/v/v ratio, respectively. For fluorescent nanocups, 9ACM was mixed with MMA in a 1:99 molar ratio. Similarly for fluorescein labeled nanocups, FoMA was mixed with MMA in a 1:99 molar ratio as well. The subsequent monomer solution was added with HEMA and DVB into the template particle mixture in a 10.1:1:6.4 9ACM (or FoMA)-MMA/HEMA/DVB volume ratio. The reaction was initiated by the addition of 1.7 mL KPS at a 3.5 mg mL^−1^ concentration in water and rigorously mixed for 5 h. The nanoparticle solution was then washed via centrifugation and suspended in DI water. To enable gas entrapment on the surface of the particles, the solution was air-dried overnight. The dry cake was suspended in 200 nm filtered DI water with 5% glucose and rigorously mixed to maximize dispersion. Coarse filtration was used to remove millimeter-sized agglomerates that did not disperse. Syringe filtration through a microporous filter was used to remove micro agglomerates when necessary.

*Nanocup Characterization*: Size distributions for nanocups were measured using DLS (ZetaSizerNano, Malvern) with a red laser. Each sample was tested with three measurements of approximately 10 runs. The correlation function for each sample was observed to be a smooth single exponential decay, indicating that there were no large (>1000 nm) particles present in the sample. Concentrations were determined via desiccation of 20 μL of sample, and weighing the resulting mass. Surface charge measurements were tested in 50 × 10^−3^ m HEPES buffer.

To confirm the size and determine the shape of the particles, we used TEM (Hitachi H-7650, Berkshire, UK) operated at 120 kV. The nanoparticles were deposited onto a Formvar supported 200 meshed grid copper grid (Electron Microscopy Sciences, Pennsylvania, USA) via air drying overnight. The images were recorded on a 2k × 2k CCD camera (Advanced Microscopy Technologies, Suffolk, UK).

*Animal Studies*: Animal experimentation was performed in accordance with UK Home Office guidelines and the United Kingdom Coordinating Committee on Cancer Research Guidelines for Welfare of Animals in Experimental Neoplasia. Female BALB/c mice were subcutaneously implanted with 2 × 10^5^ CT-26 cells and experiments commenced when tumors reached 100–300 mm^3^. A cannula was placed in the tail vein and the mouse transferred to a heated water bath. The tumor was aligned to the focal region of a conventional abdominal diagnostic ultrasound probe (C5-2, 128 element) focused to 45 mm axially with focal spot 5.2 mm by 1.2 mm. An injection of 50 μL of 5% glucose buffer was delivered and a “background” ultrasound exposure (2 MHz center frequency and 4.5 MPa pressure amplitude) sweep performed for 1 min to verify lack of cavitation. In two cases, the focus was electronically steered through ±3°, and in one case, the focus was fixed. Next, 50 μL per 0.3 mg of nanocups were injected via the cannula, and the ultrasound exposure was repeated as before either with electronic beam steering or fixed focus. Injection of nanocups and ultrasound exposure was again repeated 2 and 4 min later. Ultrasound was both delivered and cavitation detected and recorded using the same diagnostic probe connected to a Verasonics Vantage system. Custom scripting interlaced low-amplitude imaging pulses, which were received and reconstructed into B-mode images, with high-amplitude therapy pulses, which received and reconstructed into PAMs and overlaid on the B-mode images.

The study on the persistence of inertial cavitation from microbubbles and nanocups in tumors was conducted with CD-1 nude mice with subcutaneous implanted 5 × 10^6^ of SKOV-3 cells. Experiments were conducted when the tumor size reached 100–300 mm^3^. After a tail vein injection of the cavitation nuclei, the tumor was immediately exposed to ultrasound (0.5 MHz, 1.5 MPa, 0.5 Hz PRF) for at least 120 s. The ultrasound transducer had a rectangular hole cut out to fit the L11-4v diagnostic probe (4 to 11 MHz center frequency). Inertial cavitation from the nuclei was recorded and processed using the diagnostic probe (confocally aligned to the therapeutic ultrasound transducer) and PAM method mentioned above.

For antibody delivery studies, BALB/c mice bearing CT-26 tumors, see above procedure, were intravenously injected with a mixture of 150 μg of fluorescein-conjugated mouse IgG antibody and nanocups (1 mg). The tumors were exposed to ultrasound (0.5 MHz, 1.5 MPa, 0.5 Hz PRF) for a total of 12 min with the focus of the ultrasound being moved every 3 min. Mice were culled after treatment. The tumors were immediately snap-frozen in optimal cutting temperature (OCT) compound. After, 8 μm sections were acquired using a cryotome and placed onto glass slides. Sections were fixed under a coverslip in hard set immunomount (Vector labs). Endothelial and tumor cells were stained for and imaged as in reference.[5] See Tables S1 and S2 (Supporting Information) for all ultrasound equipment and exposure settings, respectively.

*Tissue Model Studies*: A tissue model was made with ultrapure agarose powder dissolved in filtered DI water with a final concentration of 1 wt%. The solution was heated to boiling point and degassed for more than 30 min, followed by cooling to approximately 40° C. A custom-built 20 mL Delrin cylindrical phantom with Mylar sheets on the front and back face had a 1.6 mm diameter stainless steel rod inserted through the center (Figure S3, Supporting Information). Agarose solution was poured into the cylindrical phantom, and allowed to cool at 4 °C overnight. Once the agarose had set, the steel rod was removed, and Tygon tubing was attached to the inlet and outlet ports. The tissue model was placed in a degassed water tank heated to 37 °C and allowed to thermally equilibrate. Prior to the introduction of the different model drug formulations, the tissue model was flushed with DI water for 3 min to remove any debris. TRITCD was used as the model drug, and was mixed with the different cavitation agents at a 0.16 mg mL^−1^ concentration. The gas fraction of SonoVue (SV) and fluorescent nanocups was matched to 0.004, which is the gas fraction of SV after a bolus injection. The gas fraction of fluorescent nanocups was estimated assuming that 20% of the nanoparticle volume was gas. Each of the model drug formulations was injected into the tissue model at a constant flow rate (300 μL min^−1^) for 30 s before exposure to 5 min of ultrasound (see Table S1 and S2, Supporting Information for all ultrasound equipment and exposure settings, respectively.). After ultrasound exposure, the tissue model was flushed with DI water, removed from the chamber, and sliced across its circular face in the direction of ultrasound propagation (Figure 2, Supporting Information). The slices were removed and placed on a glass slide for observation. A small amount of water was added to the surface of the agarose slice to remove surface artifacts. Images were taken on a Nikon TI Eclipse fluorescent microscope using the DAPI filter and tetrarhodamine (TRITC) filter sequentially. The images were stitched together to form a large composite image using Nikon AR software. ImageJ was used to analyze the images. Brightness and contrast were adjusted equally across all images.

## Supporting Information

As a service to our authors and readers, this journal provides supporting information supplied by the authors. Such materials are peer reviewed and may be re-organized for online delivery, but are not copy-edited or typeset. Technical support issues arising from supporting information (other than missing files) should be addressed to the authors.
