# Supplementary material for: Ultrasound-Propelled Nanocups for Drug Delivery
Source: Small. 2015 Aug 21;11(39):5305–14. doi: 10.1002/smll.201501322 (PMC4660885; doi:10.1002/smll.201501322)
Supplement: Supplementary file 1 — Supplementary [file smll0011-5305-sd1.pdf]

Copyright WILEY-VCH Verlag GmbH & Co. KGaA, 69469 Weinheim, Germany, 2015.

NANO small MICRO

Supporting Information

for *Small*, DOI: 10.1002/smll.201501322

Ultrasound-Propelled Nanocups for Drug Delivery

*James J. Kwan, Rachel Myers, Christian M. Coviello, Susan M. Graham, Apurva R. Shah, Eleanor Stride, Robert C. Carlisle, and Constantin C. C oussios**

Copyright WILEY-VCH Verlag GmbH & Co. KGaA, 69469 Weinheim, Germany, 2013.

Supporting Information

Title: Ultrasound-Propelled Nanocups for Drug Delivery

Authors: James J. Kwan, Rachel Myers, Christian M. Coviello, Susan M. Graham, Apurva R. Shah, Eleanor Stride, Robert C. Carlisle, Constantin C. Coussios[*]

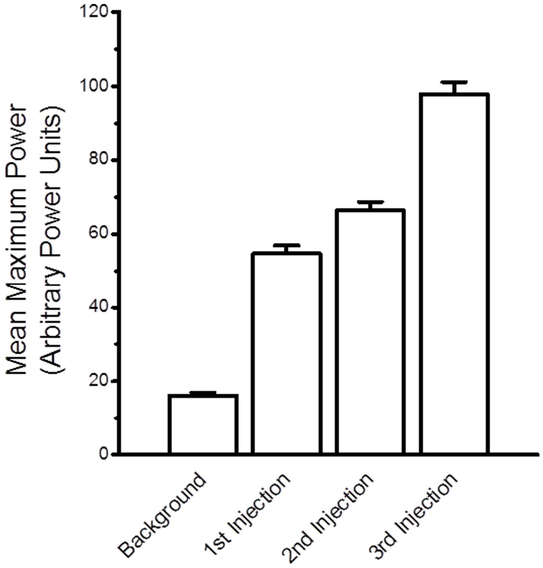

Figure S1. Evidence for accumulation of nanocups within the tumor. Averages of the mean maximum power from the passive acoustic map overlay across the entire acoustic sweep for three mice are shown. Because the values are the averages of a mean value, the error bars are shown as the standard error of the mean.

SI Section 1. Biodistribution and Pharmacokinetics

SI Secction 1.1 Methods

Animal experimentation was performed in accordance with UK Home Office guidelines and the United Kingdome Coordinating Committee on Cancer Research Guidelines for Welfare of Animals in Experimental Neoplasia. Female BALB/c mice were subcutaneously implanted with $2x10^5$ CT-26 cells and experiments commenced when tumors reached 100-300 mm^3. A cannula was placed in the tail vein and the mouse transferred to a heated water bath. To study the biodistribution and pharmacokinetics, 100 μL of fluoroscene labeled nanocups were injected at 3.1, and 6.1 mg/mL as a single bolus into the tail vein. At 5, 15, 30, and 120 minutes, 20 μL blood samples were aquired. The samples were then mixed with 180 μL 5% glucose, and centrifuged at 2000 rpm for 3 minutes. The supernatant was removed and frozen for analysis. The remaining pellet was resuspended in 190 μL 5% glucose and centrifuged. The subsequent supernatant was frozen for further analysis. All samples were stored in amber eppendorf tubes to minimize light exposure. After thawing, all samples were anaylsed with a FLUOstar Omega plate reader (BMG Labtech, Aulesbury, UK) with excitation wavelength of 490 nm and emission wavelength of 520 nm.

The organs (heart, kidneys, liver, lungs, spleen, and tumor) were harvested from euthanised mice, and were immediately frozen and stored for further analysis. After thawing, organs were lysed (E397A, Promega, Madison, WI, USA) and then homogenized (Homogenizer, Workcenter, IKA T10 Basic, Germany). A standard curve was generated by incubating homogonized control samples spiked with known amounts of labeled nanocups at 37 ºC for 30 minutes, frozen, and thawed before assaying to mimic sample preparation. All samples were measured for fluoresence using FLUOstar Omega plate reader with excitation wavelength of 490 nm and emission wavelength of 520 nm.

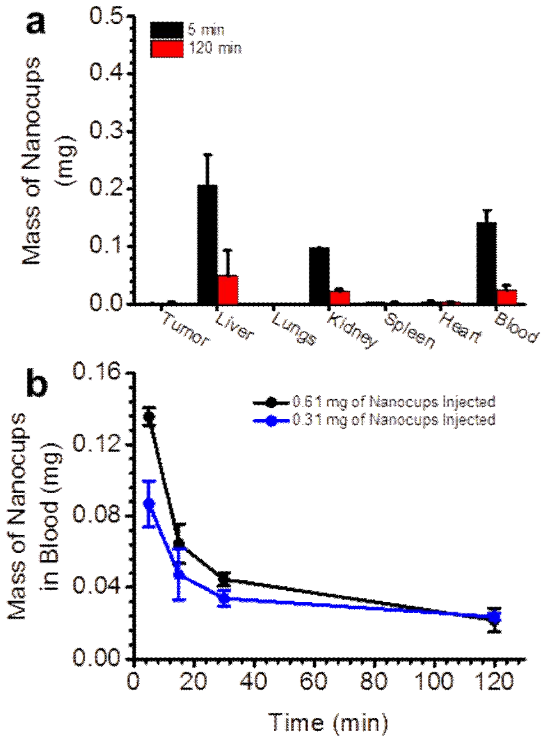

Figure S2. Biodistribution and pharmacokinetics of nanocups after tail vein injection into mice. (a) Biodistribution of fluorescein labelled nanocups from harvested organs. Measurements were taken from the total organ. (b) Circulation time of fluorescein nanocups indicating between 15 – 20% of the initial dose by 15 minutes.

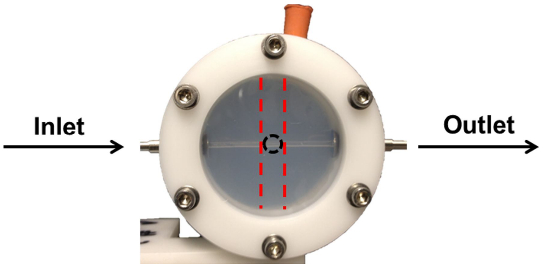

Figure S3. Image of tumor model chamber. An image of the tissue model chamber shows a 1.6 mm channel casted into agarose. The inlet, outlet, and direction of flow are also shown. The dashed circle outlines the place of focused ultrasound exposure, which is going into the page. The red dashed lines indicate where the section of agarose was sliced for imaging.

Table S1. List of acoustic equipment.

	Ultrasound Probe	Waveform Generator	PCD	RF Amplifier	Signal Amplifier
Animal Studies (Cavitation Monitoring)	C5-2, 128 element, 0.508mm pitch, (Verasonics, Inc)	N/A	N/A	N/A	N/A
Animal Studies (Cavitation Nuclei Comparison)	Spherically focused 0.5 MHz Fundamental Harmonic H107-010B (Sonic Concepts)	Agilent 33220A	L11-4v, 128 element, 0.298mm pitch (Verasonics, Inc)	E&I 1140LA	N/A
Animal Studies (Antibody Study)	Spherically focused 0.5 MHz Fundamental Harmonic H107-013D (Sonic Concepts)	Agilent 33220A	Static: 7.5 MHz SN-671678 (Panametrics)	E&I 1140LA	SR445A (Stanford Research Systems)
Tissue Mimic	Spherically focused 0.5 MHz Fundamental Harmonic H107-013D (Sonic Concepts)	Agilent 33220A	15 MHz SN-679680 (Panametrics)	E&I 1140LA	SR445A (Stanford Research Systems)

Table S2. Summary of acoustic parameters.

	Centre Frequency (MHz)	Range of Peak Rarefactional Pressures (MPa)	Duty Cycle (%)	Pulse Repetition Frequency (Hz)
Animal Study (Cavitation Monitoring)	2	4.5	0.002	4
Animal Study (Nuclei Comparison)	0.5	1.5	5	0.5
Animal Study (Antibody)	0.5	1.5	5	0.5
Tissue Mimic	1.6	2.2, 4.0	5, 1.5	50, 10
